# Genomic Adaptation of the *Lactobacillus casei* Group

**DOI:** 10.1371/journal.pone.0075073

**Published:** 2013-10-08

**Authors:** Hidehiro Toh, Kenshiro Oshima, Akiyo Nakano, Muneaki Takahata, Masaru Murakami, Takashi Takaki, Hidetoshi Nishiyama, Shizunobu Igimi, Masahira Hattori, Hidetoshi Morita

**Affiliations:** 1 Medical Institute of Bioregulation, Kyushu University, Higashi-ku, Fukuoka, Japan; 2 Graduate School of Frontier Sciences, The University of Tokyo, Kashiwa, Chiba, Japan; 3 School of Veterinary Medicine, Azabu University, Sagamihara, Kanagawa, Japan; 4 JEOL Ltd., Akishima, Tokyo, Japan; 5 Division of Biomedical Food Research, National Institute of Health Sciences, Kamiyoga, Setagaya, Tokyo, Japan; University of Strasbourg, France

## Abstract

*Lactobacillus casei*, *L. paracasei*, and *L. rhamnosus* form a closely related taxonomic group (*Lactobacillus casei* group) within the facultatively heterofermentative lactobacilli. Here, we report the complete genome sequences of *L. paracasei* JCM 8130 and *L. casei* ATCC 393, and the draft genome sequence of *L. paracasei* COM0101, all of which were isolated from daily products. Furthermore, we re-annotated the genome of *L. rhamnosus* ATCC 53103 (also known as *L. rhamnosus* GG), which we have previously reported. We confirmed that ATCC 393 is distinct from other strains previously described as *L. paracasei*. The core genome of 10 completely sequenced strains of the *L. casei* group comprised 1,682 protein-coding genes. Although extensive genome-wide synteny was found among the *L. casei* group, the genomes of ATCC 53103, JCM 8130, and ATCC 393 contained genomic islands compared with *L. paracasei* ATCC 334. Several genomic islands, including carbohydrate utilization gene clusters, were found at the same loci in the chromosomes of the *L. casei* group. The *spaCBA* pilus gene cluster, which was first identified in GG, was also found in other strains of the *L. casei* group, but several *L. paracasei* strains including COM0101 contained truncated *spaC* gene. ATCC 53103 encoded a higher number of proteins involved in carbohydrate utilization compared with intestinal lactobacilli, and extracellular adhesion proteins, several of which are absent in other strains of the *L. casei* group. In addition to previously fully sequenced *L. rhamnosus* and *L. paracasei* strains, the complete genome sequences of *L. casei* will provide valuable insights into the evolution of the *L. casei* group.

## Introduction

The genus *Lactobacillus* is the largest group of the family *Lactobacteriaceae* and contains more than 130 species. The species *Lactobacillus casei*, *L. paracasei*, and *L. rhamnosus* are phylogenetically and phenotypically closely related and are regarded together as the *Lactobacillus casei* group within the facultatively heterofermentative lactobacilli [1]. The classification and nomenclature of this group are controversial [2]–[7]. Some strains of *L. casei*, *L. paracasei*, and *L. rhamnosus* have for long been used as probiotics in a wide range of different products marketed in many countries. *L. casei* and *L. paracasei* have also been isolated from a variety of environmental habitats, including raw and fermented dairy (especially cheese) and plant materials (e.g., wine, pickle, silage, and kimchi). They are used as acid-producing starter cultures in milk fermentation as adjunct cultures for intensification and for acceleration of flavor development in bacterial-ripened cheeses. They are commonly the dominant species of nonstarter lactic acid bacteria in ripening cheese.

In the *L. casei* group, the genomes of five *L. paracasei* strains (ATCC 334, BD-II, BL23, LC2W, and Zhang) and three *L. rhamnosus* strains (ATCC 53103, Lc 705, and ATCC 8530) have been fully sequenced to date [8]–[14]. We have also previously reported the complete genome sequence of *L. rhamnosus* ATCC 53103 [15]. *L. rhamnosus* GG, the original strain of *L. rhamnosus* ATCC 53103, was isolated from a healthy human intestinal flora, and is one of the most widely used and well-documented probiotics, which confer a health benefit on the host when administered in adequate amounts [16]. It has been reported that *L. rhamnosus* GG can shorten the duration of infectious diarrhea, reduce antibiotic-associated symptoms, and alleviate food allergy and atopic dermatitis in children [16].

In this paper, we present the complete genome sequences of *L. casei* ATCC 393 and *L. paracasei* JCM 8130 (also known as ATCC 25302), which were isolated from a cheese and milk product, respectively, and the draft genome sequence of *L. paracasei* COM0101 isolated from a commercial fermented milk product. Furthermore, we re-annotated the genome of *L. rhamnosus* ATCC 53103. We then compared sequenced genomes of the *L. casei* group to gain a broader view of the genetic variability within the group. Comparison of the genome sequences of strains isolated from the human gut and dairy products can provide valuable insights into the lifestyle adaptation of the *L. casei* group.

## Materials and Methods

### Genome Sequencing

*L. paracasei* JCM 8130 and *L. casei* ATCC 393 were obtained from the Japan Collection of Microorganisms (JCM) and the American Type Culture Collection (ATCC), respectively. In this study, ten strains of putative *L. paracasei* isolated from the fermented milk product Yakult (Yakult Ltd., Japan) exhibited the same pattern by random amplification of polymorphic DNA fingerprinting [17]. We thus selected one *L. paracasei* strain designated as COM0101 for sequencing. *L. paracasei* JCM 8130, *L. casei* ATCC 393, and *L. paracasei* COM0101 were cultured in MRS (deMan, Rogosa and Sharpe) broth (Difco) at 37°C for 24 h, and the genomic DNAs were isolated and purified as previously described [18].

The genome sequences of *L. paracasei* JCM 8130, *L. casei* ATCC 393, and *L. paracasei* COM0101 were determined by the whole-genome shotgun strategy using Sanger sequencing (3730xl DNA sequencers) and 454 pyrosequencing (GS-FLX sequencers). We generated 19,200 (3.9-fold, 3730xl) and 284,003 (25.7-fold, GS-FLX) sequences from the *L. paracasei* JCM 8130 genome, 28,416 (5.9-fold, 3730xl) sequences from the *L. casei* ATCC 393 genome, and 131,707 (15.4-fold, GS-FLX) sequences from the *L. paracasei* COM0101 genome. The 454 pyrosequencing reads were assembled using the Newbler assembler software. A hybrid assembly of 454 and Sanger reads was performed using the Phred-Phrap-Consed program. Gap closing and re-sequencing of low-quality regions were conducted by Sanger sequencing to obtain the high-quality finished sequence. The overall accuracy of the finished sequence was estimated to have an error rate of <1 per 10,000 bases (Phrap score of ≥40). The deep sequencing dataset of *L. paracasei* JCM 8130 and *L. paracasei* COM0101 are deposited in the DDBJ/GenBank/EMBL Sequence Read Archive under the accession numbers DRA000955 and DRA000956, respectively.

### Informatics

An initial set of predicted protein-coding genes was identified using Glimmer 3.0 [19]. Genes consisting of <120 base pairs (bp) and those containing overlaps were eliminated. All predicted proteins were searched against a non-redundant protein database (nr, NCBI) using BLASTP with a bit-score cutoff of 60. The start codon of each protein-coding gene was manually refined from BLASTP alignments. The tRNA genes were predicted by the tRNAscan-SE [20], and the rRNA genes were detected by BLASTN search using known *Lactobacillus* rRNA sequences as queries. Protein domains were identified using HMMER with the Pfam database. Orthology across whole genomes has been determined using BLASTP reciprocal best hits in all-against-all comparisons of amino acid sequences. Two sequences were identified as highly conserved orthologs if their BLAST score ratio is more than 0.8. When two genome sequences were compared using BLASTN, non-matching regions were predicted as genomic islands. The presence of an N-terminal signal peptide sequence was predicted using the SignalP [21]. Clustered regularly interspaced short palindromic repeats (CRISPR) were predicted using the CRISPRFinder [22]. Draft genome sequences of *L. rhamnosus* ATCC 21052 (accession no. AFZY01000000), *L. rhamnosus* HN001 (ABWJ00000000), *L. rhamnosus* LMS2-1 (ACIZ00000000), *L. paracasei* 8700∶2 (ABQV00000000), and *L. casei* (*zeae*) KCTC 3804 (BACQ01000000) were obtained from GenBank.

The complete genome sequences of *L. paracasei* JCM 8130, *L. casei* ATCC 393, and *L. rhamnosus* ATCC 53103 are deposited in the DDBJ/GenBank/EMBL database under the accession numbers AP012541–AP012543, AP012544–AP012546, and AP011548, respectively. The draft genome sequence of COM0101 has been deposited in public database under the accession numbers BAGT01000001–BAGT01000184.

## Results and Discussion

### Comparative Genome Analysis within the *L. casei* Group

**Figure 1:**
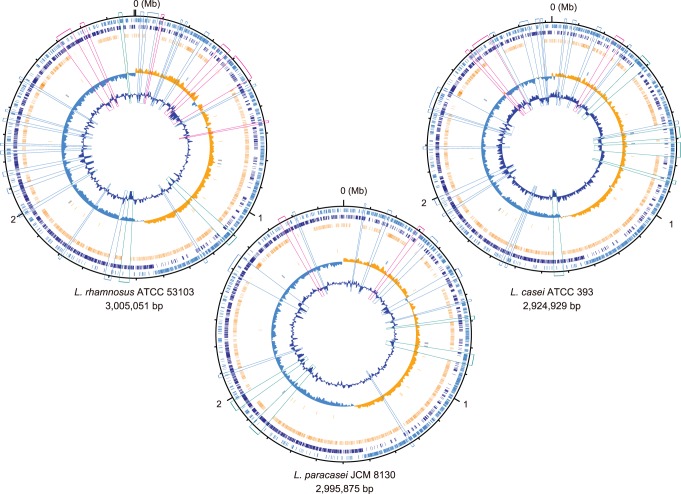
Circular representations of the chromosomes of *L. rhamnosus* ATCC 53103, *L. paracasei* JCM 8130, and *L. casei* ATCC 393. From the outside: circles 1 and 2 of the chromosome show the positions of protein-coding genes on the positive and negative strands, respectively. Circle 3 shows the positions of protein-coding genes that are shared among the 10 completely sequenced genomes of the *L. casei* group. Circle 4 shows the positions of tRNA genes (orange) and rRNA genes (blue). Circle 5 shows a plot of GC skew [(G − C)/(G+C); orange indicates values >0; blue indicates values <0]. Circle 6 shows a plot of G+C content (outward: higher values than the average). The genomic islands in each strain are boxed: regions including carbohydrate utilization gene cluster (pink), prophage-like regions (green), and the others (blue).

**Figure 2:**
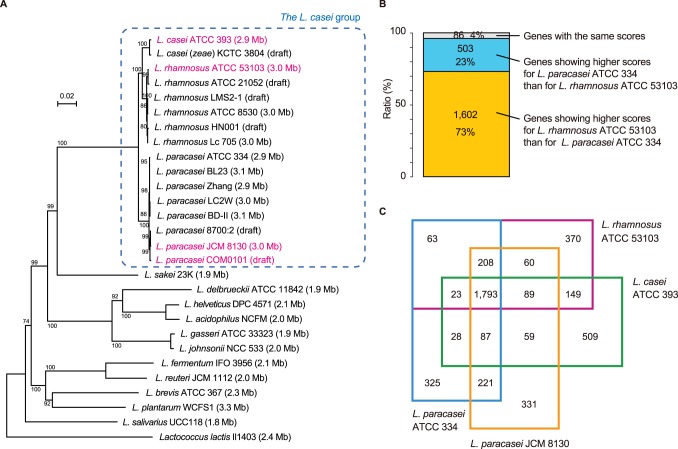
Genome-based phylogenetic analysis of the *L. casei* group. (**A**) Phylogenetic relationships between the genomes of sequenced lactobacilli inferred from 34 concatenated ribosomal protein amino acid sequences. The scale bar represents an evolutionary distance. Sequences were aligned with ClustalW with a bootstrap trial of 1,000 and bootstrap values (%) are indicated at the nodes. An unrooted tree was generated using NJplot. The chromosome size is shown in parentheses. (**B**) Three-way comparisons between *L. casei* ATCC 393 with *L. rhamnosus* ATCC 53103 and *L. paracasei* ATCC 334. The 2,191 genes shared by the three strains were classified into three categories on the basis of the BLAST score ratio analysis [23]. (**C**) Venn diagram comparing the gene inventories of four strains of the *L. casei* group. Data resulted from reciprocal BLASTP analysis. The numbers of shared and unique genes are shown.

We first re-annotated the genome of *L. rhamnosus* ATCC 53103, which we previously reported in the short paper [15]. Next, we determined and annotated the complete genome sequences of *L. paracasei* JCM 8130 and *L. casei* ATCC 393. The genome of *L. paracasei* JCM 8130 consists of a circular chromosome of 2,995,875 bp and two plasmids, and that of *L. casei* ATCC 393 consists of a circular chromosome of 2,924,929 bp and two plasmids (Fig. 1). The chromosomes of *L. paracasei* JCM 8130 and *L. casei* ATCC 393 contained 2,848 and 2,737 predicted protein-coding genes, respectively. The larger plasmid (27 kilobases [kb]) of ATCC 393 shared 14 genes, such as beta-galactosidase and cystathionine beta-synthase, with a 65-kb plasmid (accession no. FM179324) of *L. rhamnosus* Lc 705 (Fig. S1), thus indicating that both plasmids may be derived from the same origin. Furthermore, we generated a draft genome sequence of *L. paracasei* COM0101 that consists of 184 contigs (>500 bp) with a total length of 3,003,364 bp. The COM0101 genome contained 2,767 predicted protein-coding genes. One of the highly redundant contigs contained a gene for plasmid replication protein that showed 100% amino acid identity with that of *L. paracasei* strains, indicating that the COM0101 genome probably has at least one plasmid. Their chromosome sizes (2.9–3.0 megabases [Mb]) were among the largest group in the *Lactobacillus* genomes, with an average size of 1.8–2.0 Mb (Fig. 2A). General features of these genomes are summarized in Table S1.

We constructed a phylogenetic tree for concatenated sequences of ribosomal proteins from sequenced *Lactobacillus* (Fig. 2A). *L. casei* ATCC 393 and the *L. casei*–*paracasei* phylum were found to form a distinct clade within the *L. casei* group, and *L. casei* ATCC 393 was shown to be closer to *L. casei* (*zeae*) KCTC 3804. A three-way comparison between the genomes of *L. casei* ATCC 393, *L. rhamnosus* ATCC 53103, and *L. paracasei* ATCC 334 using the BLAST score ratio analysis [23] revealed a greater number of proteins in *L. casei* ATCC 393 showing a high score for *L. rhamnosus* ATCC 53103 than those showing a high score for *L. paracasei* ATCC 334 (Fig. 2B). Moreover, *L. casei* ATCC 393 shared more genes with *L. rhamnosus* ATCC 53103 than with *L. paracasei* ATCC 334 (Fig. 2C). We thus found that *L. casei* ATCC 393 is more closely related to *L. rhamnosus* strains than to *L. paracasei* strains based on the phylogeny, overall protein similarities, and number of shared genes. This result supports the previous reports that *L. casei* ATCC 393 is distinct from other strains previously described as *L. paracasei* [2], [3], [5], [6]. Furthermore, we also constructed a multi-locus sequence typing (MLST)-based phylogenetic tree [24] for *L. paracasei* strains (Fig. S2A), showing that COM0101 shares the same MLST lineage with BL23, LC2W, and BD-II. Moreover, COM0101 shared more genes with BL23 than with ATCC 334 and JCM 8130 (Fig. S2B). Thus, COM0101 is phylogenetically closely related to BL23, LC2W, and BD-II in *L. paracasei* strains.

**Figure 3:**
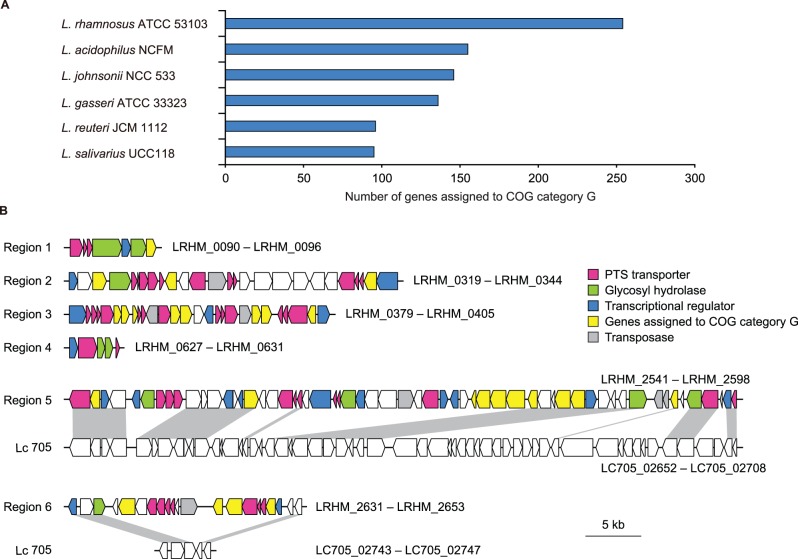
Abundance of genes related to carbohydrate transport and metabolism in *L. rhamnosus* ATCC 53103. (**A**) Comparative analysis by functional categories of the gene repertoires of sequenced intestinal lactobacilli. The number of genes assigned to COG category G in each genome is shown. (**B**) Carbohydrate utilization gene clusters of *L. rhamnosus* ATCC 53103. Genes and their orientations are depicted with arrows. Regions -5 and -6 are compared with the corresponding genomic locations in *L. rhamnosus* Lc 705. Gray bars indicate orthologous regions.

We compared the genomes of *L. rhamnosus* ATCC 53103, *L. paracasei* JCM 8130, *L. casei* ATCC 393, and *L. paracasei* ATCC 334 (Fig. 2C). Thus, 1,793 genes were common to the four strains, and a total of 4,315 ortholog clusters were assigned to the pan-genome of the four strains. Of the 1,793 core genes, 1,682 (94%) were also conserved among the other six completely sequenced strains (BD-II, BL23, LC2W, Zhang, Lc 705, and ATCC 8530) of the *L. casei* group. Broadbent *et al*. (2012) showed that 1,715 protein-coding genes were common to 17 sequenced *L. casei* strains [25]. These results suggest that approximately 1,700 genes constitute the core genome of the *L. casei* group, likely inherited from their common ancestor. All dispensable protein-coding genes, which were found in one or more but not all the 10 completely sequenced strains of the *L. casei* group, were functionally classified based on the clusters of orthologous groups from the NCBI COGs database, and the gene repertoires were compared (Fig. S3). There was a considerable difference in the number of genes assigned to COG category G (carbohydrate transport and metabolism) and category L (replication, recombination, and repair) among the strains. *L. rhamnosus* strains had a lower number of genes assigned to COG category L because the *L. rhamnosus* genomes contained a lower number of transposase genes compared with the other strains, suggesting that insertion element-mediated genome diversification is less frequent in *L. rhamnosus* strains. In contrast, *L. paracasei* JCM 8130 and *L. casei* ATCC 393 contained a higher number of transposase genes. Most of the genes assigned to COG category G were encoded in hypervariable regions in the genomes of the *L. casei* group (described later). We next classified all protein-coding genes of *L. rhamnosus* ATCC 53103 and sequenced intestinal lactobacilli on the basis of the COGs database (Fig. 3A). *L. rhamnosus* ATCC 53103 contained a higher number of genes assigned to COG category G compared with intestinal lactobacilli. The abundance of genes related to carbohydrate transport and metabolism in *L. rhamnosus* ATCC 53103 may contribute to the wide variety of qualities in this strain compared with other probiotics.

Bacteriocins are small antimicrobial peptides produced widely by lactic acid bacteria. The *L. rhamnosus* ATCC 53103 genome encoded the bacteriocin gene cluster (LRHM_2289 to LRHM_2312), which contained genes encoding the two-component sensor and regulator, four bacteriocin immunity proteins, ATP-binding cassette (ABC) transporter with the proteolytic domain, and small peptides. The cluster was conserved in the genomes of the *L. casei* group, but in the corresponding region of *L. casei* ATCC 393, a gene for bacteriocin ABC transporter was interrupted by transposase (LBCZ_2129 to LBCZ_2133) and genes for immunity proteins were absent, suggesting that *L. casei* ATCC 393 may not be able to produce bacteriocin.

CRISPRs, along with their associated *cas* genes, are known to constitute a defense system against the propagation of phages and plasmids; these were observed in the genomes of a number of lactic acid bacteria [26]. *L. rhamnosus* ATCC 53103 contained a CRISPR region (2,260,261–2,261,880) and four CRISPR-associated genes (LRGG_2116 to LRGG_2119). The 36-bp-long sequence was present 25 times and separated by 30-bp unique spacer sequences. It has been reported that two distinct types (Lsal1 and Ldbu1) of CRISPR loci were identified in the *L. casei* genomes [25]. *L. casei* strains BD-II, BL23, LC2W, and Zhang also have an Lsal1-type CRISPR region at the same locus on the chromosome, suggesting that the ancestral strain of the *L. casei* group had encoded a CRISPR region. However, the 36-bp repeat sequence of the four *L. casei* strains differs by two bases from that of *L. rhamnosus* ATCC 53103, and the number of the repeat sequences was different (17–22) among these strains. COM0101 has the orthologs of the four CRISPR-associated genes, indicating that COM0101 also may have a CRISPR region. In contrast, *L. paracasei* JCM 8130, *L. casei* ATCC 393, *L. rhamnosus* Lc 705, and *L. rhamnosus* ATCC 8530 had no CRISPR, suggesting that these strains may have lost a CRISPR region during adaptation to their environment where phage detection is not essential.

### Genomic Islands

Whole-genome alignment showed a high level of synteny among the strains of the *L. casei* group (Fig. S4). A previous report showed that there was a high degree of synteny among the genomes of 17 *L. casei* strains [25]. These results indicate that strains of the *L. casei* group have a stable genome structure. However, each genome contained specific genes, many of which were grouped in clusters as genomic islands (GIs). It has been reported that the comparison of the genomes of *L. paracasei* ATCC 334 and BL23 revealed 12 and 19 GIs (>5 kb) in ATCC 334 and BL23, respectively [27]. Similarly, we identified 26 GIs (>5 kb) in *L. rhamnosus* ATCC 53103 that were not conserved in *L. paracasei* ATCC 334 (a cheese isolate) (Table 1, Fig. 1). The 26 genomic islands of *L. rhamnosus* ATCC 53103 included six carbohydrate utilization gene clusters (regions −1 to −6), four of which were completely or partially present in *L. paracasei* BL23, whose ecological origin is unclear. This result supports the previous findings that cheese isolates, including *L. paracasei* ATCC 334, have undergone significant gene decay, including loss of many genes involved in carbohydrate utilization [25], [27]. Thus, *L. paracasei* ATCC 334 contains a lower number of genes related to carbohydrate transport and metabolism compared with the other sequenced *L. paracasei* strains (Fig. S3). In probiotic lactobacilli, horizontal gene transfer played an important role in shaping the common ancestor [28]. Such acquisition of new genes can expand a bacterium’s potential for adaptation to a new niche. The common ancestor of *L. rhamnosus* ATCC 53103 and *L. paracasei* ATCC 334 seems to have acquired carbohydrate utilization gene clusters via lateral gene transfer. These carbohydrate utilization gene clusters may have provided adaptive features to some strains including ATCC 53103 for their survival and proliferation in the human intestine. In contrast, these carbohydrate utilization gene clusters may have been lost in the lineage to ATCC 334 during its adaptation to the cheese environment.

**Table 1:** Genomic islands in *L. rhamnosus* ATCC 53103, *L. paracasei* JCM 8130, and *L. casei* ATCC 393.

*L. rhamnosus* ATCC 53103			*L. paracasei* JCM 8130			*L. casei* ATCC 393		
Locus	Size (kb)	Product description	Locus	Size (kb)	Product description	Locus	Size (kb)	Product description
LRHM_0019– LRHM_0031	12.5	ammonium transporter protein,hypothetical protein	LBPC_0071– LBPC_0078	6.1	conserved hypothetical protein	LBCZ_0041– LBCZ_0051	8.3	hypothetical protein, transposase
LRHM_0044– LRHM_0073	39.7	fibronectin-binding protein, beta-glucuronidase, 2-dehydro-3-deoxygluconokinase, mannonate dehydratase, fructuronate reductase	LBPC_0157– LBPC_0170	15.5	conserved hypothetical protein	LBCZ_0065– LBCZ_0076	12.6	transposase, conserved hypothetical protein
LRHM_0086– LRHM_0096	10.5	carbohydrate utilization gene cluster (region-1)	LBPC_0276– LBPC_0297	23.8	carbohydrate utilization gene cluster	LBCZ_0159– LBCZ_0174	15.7	myo-inositol catabolism protein
LRHM_0149– LRHM_0156	6.1	carbohydrate transporter, two-componentsystem	LBPC_0331– LBPC_0359	30.6	PTS transporter, amino acid ABC transporter	LBCZ_0223– LBCZ_0252	33.8	carbohydrate utilization gene cluster
LRHM_0172– LRHM_0177	6.6	taurine ABC transporter	LBPC_0470– LBPC_0499	33.4	hypothetical protein	LBCZ_0277– LBCZ_0286	8.9	conserved hypothetical protein
LRHM_0256– LRHM_0268	14.8	myo-inositol catabolism protein	LBPC_0579– LBPC_0584	6.7	PTS transporter, 6-phospho-beta-galactosidase	LBCZ_0338– LBCZ_0388	41.1	prophage region I
LRHM_0319– LRHM_0350	34.6	carbohydrate utilization gene cluster (region-2)	LBPC_0636– LBPC_0648	12.1	prophage region I	LBCZ_0605– LBCZ_0617	11.9	hypothetical protein
LRHM_0376– LRHM_0466	97.8	carbohydrate utilization gene cluster (region-3), amino acid ABC transporter, beta-N-acetylglucosaminidase, N-acylamino acid racemase, cell surface protein, transposase	LBPC_0763– LBPC_0817	41.2	prophage region II	LBCZ_0620– LBCZ_0675	39.9	prophage region II
LRHM_0493– LRHM_0500	8.0	hypothetical protein	LBPC_1168– LBPC_1176	9.2	conserved hypothetical protein	LBCZ_0685– LBCZ_0742	54.2	prophage region III
LRHM_0624– LRHM_0631	7.3	carbohydrate utilization gene cluster (region-4)	LBPC_1739– LBPC_1789	42.7	prophage region III	LBCZ_0821– LBCZ_0832	13.4	prophage region IV
LRHM_1038– LRHM_1090	39.7	prophage region I	LBPC_1864– LBPC_1906	36.7	prophage region IV	LBCZ_1343– LBCZ_1372	36.1	prophage region V
LRHM_1192– LRHM_1199	10.4	amino acid transporter, hypothetical protein	LBPC_1988– LBPC_1998	10.9	glycosyltransferase, transposase	LBCZ_1552– LBCZ_1559	7.8	truncated formate C-acetyltransferase, transcriptional regulator
LRHM_1455– LRHM_1484	36.5	prophage region II	LBPC_2364– LBPC_2427	70.9	glycosyltransferase, cell surface protein, conserved hypothetical protein	LBCZ_1571– LBCZ_1577	9.9	putative cell surface protein
LRHM_1518– LRHM_1530	24.9	cell surface protein, glycosyltransferase	LBPC_2603– LBPC_2630	27.3	carbohydrate utilization gene cluster	LBCZ_1817– LBCZ_1825	9.7	glycosyltransferase
LRHM_1699– LRHM_1703	7.7	cell surface protein	LBPC_2661– LBPC_2670	8.4	putative cell surface protein, transposase	LBCZ_1857– LBCZ_1870	23.6	conserved hypothetical protein
LRHM_1877– LRHM_1891	13.2	conserved hypothetical protein, transposase				LBCZ_2040– LBCZ_2046	9.2	conserved hypothetical protein
LRHM_1959– LRHM_1977	19.3	glycosyltransferase				LBCZ_2167– LBCZ_2179	12.2	conserved hypothetical protein, ABC transporter
LRHM_2012– LRHM_2019	16.4	conserved hypothetical protein				LBCZ_2185– LBCZ_2247	81.7	putative cell surface protein, conjugative transposon protein
LRHM_2085– LRHM_2097	12.3	conserved hypothetical protein				LBCZ_2402– LBCZ_2414	12.5	carbohydrate utilization gene cluster
LRHM_2115– LRHM_2119	8.3	CRISPR-associated protein				LBCZ_2437– LBCZ_2492	66.7	putative cell surface protein, carbohydrate utilization gene cluster
LRHM_2193– LRHM_2198	11.8	cell surface protein, glycosyltransferase				LBCZ_2499– LBCZ_2517	21.1	transposase, conserved hypothetical protein
LRHM_2223– LRHM_2230	7.3	multidrug ABC transporter, hypothetical protein				LBCZ_2616– LBCZ_2643	31.3	carbohydrate utilization gene cluster, transposase
LRHM_2351– LRHM_2356	8.1	multidrug ABC transporter				LBCZ_2678– LBCZ_2694	15.0	transposase
LRHM_2545– LRHM_2597	57.7	carbohydrate utilization gene cluster (region-5)				LBCZ_2698– LBCZ_2704	7.6	PTS transporter
LRHM_2635– LRHM_2651	15.4	carbohydrate utilization gene cluster (region-6)						
LRHM_2779– LRHM_2793	12.5	prophage region III						

Similarly, compared with *L. paracasei* ATCC 334, 15 and 24 GIs were found in *L. paracasei* JCM 8130 and *L. casei* ATCC 393, respectively (Table 1, Fig. 1). Of these GIs, 6 (JCM 8130) and 10 (ATCC 393) were found at the same loci with those of *L. rhamnosus* ATCC 53103. A comparative genome hybridization in 22 *L. casei* strains isolated from various habitats has revealed 25 hypervariable regions [27], of which 11 were found at the same loci of the GIs in *L. rhamnosus* ATCC 53103. Thus, these results suggest that the chromosomes of the *L. casei* group contain several hypervariable regions at the same loci.

The six carbohydrate utilization gene clusters of *L. rhamnosus* ATCC 53103 contained the genes for phosphoenolpyruvate-carbohydrate phosphotransferase (PTS)-type transporter systems, glycosyl hydrolases, transcriptional regulators, and other carbohydrate-related proteins (Fig. 3B). *L. rhamnosus* ATCC 53103 encoded 28 complete PTS-type transporter systems, 11 of which were encoded adjacent to genes for glycosyl hydrolase and transcriptional regulator, thereby allowing localized transcriptional control. The organization (carbohydrate transporter, glycosyl hydrolase, and transcriptional regulator) is reminiscent of the many clusters found in *Bifidobacterium longum* [29].

Six of the 26 GIs of *L. rhamnosus* ATCC 53103 overlapped with all the hypervariable regions among the sequenced *L. rhamnosus* strains (ATCC 53103, Lc 705, ATCC 8530, ATCC 2105, HN001, and LMS2-1). Three of the six hypervariable regions were prophage-like regions (LRHM_1038 to LRHM_1090, LRHM_1455 to LRHM_1475, and LRHM_2779 to LRHM_2794 in ATCC 53103). The other three regions corresponded to regions containing carbohydrate utilization gene clusters (regions -3, -5, and -6), indicating that *L. rhamnosus* strains show flexibility in sugar utilization. Two of the five PTS-type transporter systems in region-5 and two in region-6 were missing in Lc 705, ATCC 8530, and LMS2-1 strains (Fig. 3B). Comparative genomic hybridization analyses have showed that the region corresponding to regions -5 and -6 contains an overrepresentation of genes involved in carbohydrate utilization and transcriptional regulation in 22 *L. casei* strains [27]. Taken together, the region corresponding to regions -5 and -6 in the genomes of the *L. casei* group may be required to fine-tune its ability to utilize carbohydrates.

### Extracellular Components

Another group has also determined the complete genome sequence of *L. rhamnosus* GG, and revealed the presence of the SpaCBA pili on the cell surface of *L. rhamnosus* GG [9]. SpaA is a backbone-forming major pilin, SpaB is a minor pilin, and SpaC located at the pilus tip is essential for the mucus adherence of *L. rhamnosus* GG [9], [30]. The *spaCBA* genes are encoded in the largest GI (LRHM_0376 to LRHM_0466) in *L. rhamnosus* ATCC 53103 (Fig. S5). The *L. paracasei* Zhang, *L. paracasei* BL23, and *L. paracasei* ATCC 334 genomes also encode the *spaCBA* genes (Fig. S5). In contrast, *L. casei* ATCC 393 completely lacks the *spaCBA* genes. The *spaCBA* genes were also encoded in *L. paracasei* COM0101, but the *spaC* gene was truncated by a nonsense mutation [25] (Fig. S5), which probably encodes a non-functional protein. Douillard *et al*., (2013) clearly showed that the *L. paracasei* strain isolated from Yakult produced no pilus structures by an immunoelectron microscopy using immunogold staining [31]. It has been reported that the adhesion capacity of *L. rhamnosus* GG to Caco-2 cells and intestinal mucus was approximately 10 times that of strain Shirota, which was obtained from Yakult [32]. This may be because *L. rhamnosus* GG encodes the intact SpaCBA and *L. paracasei* COM0101 encodes truncated SpaC. Furthermore, *L. paracasei* JCM 8130, *L. paracasei* BD-II, and *L. paracasei* LC2W also contained truncated *spaC* gene (Fig. S5), and *L. rhamnosus* Lc 705 and ATCC 8530 completely lacked the *spaCBA* genes. The *spaCBA* genes have been found only in the *L. casei* group to date. Because different lineages in *L. casei* strains contained the *spaCBA* genes, it has been suggested that the *spaCBA* genes were not recently acquired [25]. It could thus be speculated that the ancestral strain of the *L. casei* group had encoded the intact *spaCBA* genes and then *spaCBA* may have been lost or disrupted in certain strains of the *L. casei* group.

**Figure 4:**
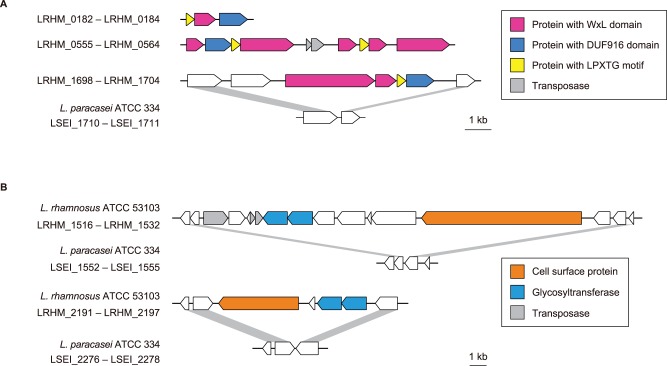
Gene clusters encoding cell surface proteins in *L. rhamnosus* ATCC 53103. (**A**) WxL clusters. (**B**) Putative glycosylated cell-surface protein clusters. Genes and their orientations are depicted with arrows. Gray bars indicate orthologous regions between *L. rhamnosus* ATCC 53103 and *L. paracasei* ATCC 334.

*L. rhamnosus* ATCC 53103 had three gene clusters encoding proteins with a C-terminal WxL domain (Fig. 4A). The WxL domain is conserved in the surface proteins in low-GC gram-positive bacteria [33] and attaches to the peptidoglycan on the cell surface [34]. The WxL protein cluster was not found in other sequenced intestinal lactobacilli. The proteins with the WxL domain were present together with the proteins containing the DUF916 domain (PF06030) of unknown function and the small proteins with the LPXTG-like sorting motif, and their gene organizations were similar to that in *L. plantarum* WCFS1 [35]. Of the three WxL protein clusters, one (LRHM_1699 to LRHM_1702) was not conserved in the sequenced *L. paracasei* strains (Fig. 4A, Table 2). There were 14 genes encoding proteins that had both a signal sequence for secretion and an LPXTG-type motif for covalent anchoring to the peptidoglycan matrix (Table 2), and these proteins can be cleaved by sortase. The protein LRHM_1529 was composed of 3,275 amino acid residues, representing the largest protein in this genome, and it contained imperfect repeats consisting of serine, alanine, and aspartic acid. This serine-rich motif has been found in the extracellular proteins in the genomes of other gram-positive bacteria such as *L. plantarum*, *L. johnsonii*, and *Streptococcus pneumoniae* [29], [36], [37]. The protein LRHM_1529 was encoded in the region (LRHM_1518 to LRHM_1530), which contained two glycosyltransferase genes (Fig. 4B). It has been suggested that glycosyltransferase, encoded by the adjacent genes, caused O-linked glycosylations on the serines in the putative cell surface protein, thus producing mucin-like structures [36]. Similarly, the protein LRHM_2193 had an LPXTG-type motif, and it contained imperfect repeats consisting of serine and alanine and two adjacent glycosyltransferase genes (Fig. 4B). Thus, LRHM_1529 and LRHM_2193 could encode glycosylated cell-surface adhesives. The protein LRHM_1797 (2,357 amino acids) plays an important modulating role in adhesion to intestinal epithelial cells and biofilm formation [38]. These genes (LRHM_1529, LRHM_1797, and LRHM_2193) were absent in the sequenced *L. paracasei* strains. The presence of a variety of the cell surface adherence proteins could contribute to the probiotic properties of *L. rhamnosus* ATCC 53103.

**Table 2:** Putative cell surface adherence proteins of *L. rhamnosus* ATCC 53103.

Locus	Size (aa)	Contained domain	SignalP	*L. paracasei* ATCC 334	*L. paracasei* BL23	*L. paracasei*Zhang	*L. paracasei*JCM 8130	*L. casei* ATCC 393	*L. rhamnosus*Lc 705	*L. rhamnosus* ATCC 8530
LRHM_0051	1,492	fibronectin-binding	+	−	−	−	−	−	+	+
LRHM_0182	106	LPXTG	+	−	−	−	−	−	+	+
LRHM_0183	268	WxL	+	+	+	+	+	+	+	+
LRHM_0184	359	DUF916	+	+	+	+	+	+	+	+
LRHM_0426	334	LPXTG (SpaA)	+	−	+	+	+	−	−	−
LRHM_0427	241	LPXTG (SpaB)	+	−	+	+	+	−	−	−
LRHM_0428	895	LPXTG (SpaC)	+	+	+	+	−	−	−	−
LRHM_0555	220	WxL1	+	+	+	+	+	+	+	+
LRHM_0556	340	DUF916	+	+	+	+	+	+	+	+
LRHM_0557	118	LPXTG	+	+	+	+	+	+	+	+
LRHM_0558	688	WxL2	+	−	+	+	−	+	+	+
LRHM_0561	238	WxL1	+	+	+	+	+	−	+	+
LRHM_0562	124	LPXTG	+	+	+	+	+	−	+	+
LRHM_0563	229	WxL1	+	+	+	+	+	−	+	+
LRHM_0564	679	WxL2	+	+	+	+	+	−	+	+
LRHM_1138	401	LPXTG	−	+	+	+	+	+	+	+
LRHM_1331	213	LysM	−	+	+	+	+	+	+	+
LRHM_1393	567	fibronectin-binding	−	+	+	+	+	+	+	+
LRHM_1528	913	Ig-like fold	+	−	−	−	−	+	+	+
LRHM_1529	3,275	LPXTG	+	−	−	−	−	+	+	+
LRHM_1699	351	DUF916	+	−	−	−	−	+	+	+
LRHM_1700	114	LPXTG	+	−	−	−	−	−	+	+
LRHM_1701	262	WxL	+	−	–	−	−	+	+	+
LRHM_1702	1,131	WxL	−	−	−	−	−	+	+	+
LRHM_1797	2,357	LPXTG	−	−	−	−	−	+	+	+
LRHM_2006	1,561	LPXTG	+	−	−	−	−	−	+	−
LRHM_2185	1,973	LPXTG	+	+	+	+	+	+	+	+
LRHM_2193	1,653	LPXTG	−	−	−	−	−	+	+	+
LRHM_2248	388	LPXTG, mucin-binding domain	−	+	+	+	+	+	+	+
LRHM_2279	517	LPXTG (SpaD)	+	+	+	+	+	−	+	+
LRHM_2281	983	LPXTG (SpaF)	+	+	+	+	+	−	+	+
LRHM_2626	1,494	LPXTG	+	−	−	−	−	−	+	+
LRHM_2815	2,603	LPXTG	+	+	+	+	+	−	+	+

* ‘+’ indicates that the orthologous gene is present, and ‘−’ indicates that the orthologous gene is absent.

## Conclusions

We determined the complete genome sequences of *L. paracasei* JCM 8130 and *L. casei* ATCC 393, and the draft genome sequence of *L. paracasei* COM0101. Furthermore, we re-annotated the genome of *L. rhamnosus* ATCC 53103. We confirmed that *L. casei* ATCC 393 is distinct from the *L. paracasei* strains previously. Comparative genome analysis revealed 1,682 core genes and genome-wide synteny in the *L. casei* group. Chromosomes of the *L. casei* group contained GIs, many of which are also found at the same loci, suggesting that the chromosomes of the *L. casei* group contain several hypervariable regions at the same loci, which may contribute to the adaptation to each ecological niche. The *spaCBA* pilus gene cluster, which was first identified in *L. rhamnosus* GG, was also found in other strains of the *L. casei* group, but several *L. paracasei* strains including COM0101 contained truncated *spaC* gene. *L. rhamnosus* ATCC 53103 encodes SpaCBA pili, proteins with WxL domain, two glycosylated cell-surface adhesives, and several large proteins with the LPXTG motif. The complete genome sequences of *L. rhamnosus*, *L. paracasei*, and *L. casei* will provide a framework that will help understand the genomic differences between strains within the *L. casei* group.

## Supporting Information

Figure S1 **Linear representations of the plasmids of** ***L. casei*** **393 and of** ***L. rhamnosus*** **Lc 705.** Genes and their orientations are depicted with arrows. Several lines connect orthologs with the following colors: red, genes sharing over 95% amino acid identity; orange, genes sharing 70–95% amino acid identity; blue, transposase genes; and green, partially conserved genes.
(EPS)

Figure S2 **Genetic relationships among** ***L. paracasei*** **strains as defined by multilocus sequence typing. (A)** Concatenated sequences of five MLST loci (*ftsZ*, *metRS*, *mutL*, *pgm*, and *polA*) were analyzed as described previously [24]. **(B)** Venn diagram comparing the gene inventories of four *L. paracasei* strains. Data resulted from reciprocal BLASTP analysis. The numbers of shared and unique genes are shown.
(EPS)

Figure S3 **COG classification of dispensable protein-coding genes of the** ***L. casei*** **group.**
(EPS)

Figure S4 **Synteny between the chromosomes in the** ***L. casei*** **group.** Each plot point represents reciprocal best matches by BLASTP comparisons between orthologs.
(EPS)

Figure S5 **The** ***spaCBA*** **pili cluster arrangement.** Genes and their orientations are depicted with arrows.
(EPS)

Table S1 **General genomic features of strains sequenced in this study.**
(PDF)
