# Supplementary material for: Genomic Adaptation of the Lactobacillus casei Group
Source: PLoS One. 2013 Oct 8;8(10):e75073. doi: 10.1371/journal.pone.0075073 (PMC3792948; doi:10.1371/journal.pone.0075073)
Supplement: Table S1 — General genomic features of strains sequenced in this study. (PDF) [file pone.0075073.s006.pdf]

Table S1. General genomic features of strains sequenced in this study

	L. rhamnosus ATCC 53103	*L. paracasei* JCM 8130	*L. casei* ATCC 393	*L. paracasei* COM0101*
Chromosome size (bp)	3,005,051	2,995,875	2,924,929	3,003,364
GC content (%)	46.7	46.6	47.9	46.3
Protein-coding gene	2,755	2,848	2,737	2,767
Assigned function	1,872	1,915	1,834	-
Conserved hypothetical	720	736	589	-
Unknown function	70	69	137	-
Phage related	93	128	177	-
rRNA operon	5	5	5	-
tRNA gene	56	61	58	-
Plasmid	None	2 (11.2 kb, 10.8 kb)	2 (26.7 kb, 1.3 kb)	-
Isolation site	human gut	milk product	cheese	milk product

* The draft sequence consists of 184 contigs.
